# Evaluation of the Safety and Efficacy of Thulium Fiber Laser Lithotripsy for Ureteric Stones During Semi-rigid Ureteroscopy

**DOI:** 10.7759/cureus.105755

**Published:** 2026-03-24

**Authors:** Abhay Mahajan, Sai Swaroop Yamajala, Sumeeta Mahajan, Girish Chirlikar

**Affiliations:** 1 Urology, Sai Urology Hospital, Aurangabad, IND; 2 Anesthesia, Sai Urology Hospital, Aurangabad, IND; 3 Radiology, Sai Urology Hospital, Aurangabad, IND

**Keywords:** laser treatment, stone lithotripsy, thulium fiber laser, ureteric stone, ureteroscopy

## Abstract

Introduction: Semi-rigid ureteroscopy is commonly performed to manage ureteric stones, and thulium fiber laser (TFL) has emerged as the preferred modality for stone lithotripsy. We evaluated the safety and efficacy of TFL in the ureter during treatment of ureteric calculi.

Methods: We retrospectively evaluated 124 consecutive patients who underwent TFL lithotripsy for ureteric stones during semi-rigid ureteroscopy. Preoperatively, the patients were evaluated using urine culture and non-contrast computed tomography. The stone size, volume, and Hounsfield units (HU) were recorded. Intraoperative parameters, including visibility, bleeding, and stone retropulsion, were assessed. Postoperative complications such as hematuria, fever, sepsis, and residual stones were evaluated. The stone-free rate was assessed using ultrasonography and kidney, ureter, and bladder (KUB) radiography.

Results: Mean stone size, volume, and HU were 10.2 mm, 237.7 mm^3^, and 962, respectively. The total power was 6 watts for the majority of the patients. Bleeding was observed in two (1.61%) patients; vision was rated as good in 94 (75.8%) cases, and stone retropulsion occurred in four (3.22%) patients. Stone ablation efficacy and ablation speed were 9.32 J/mm^3^ and 0.23 mm^3^/s, respectively. Satava complications were Grade I in two (1.16%) patients, Grade IIa in four (3.22%) patients, and Grade IIb in one (0.80%) patient. According to the Clavien-Dindo classification, complications were Grade I in 12 (9.67%) patients, Grade II in three (2.41%) patients, and Grade III in one (1.61%) patient. The stone-free rate was 98%. During follow-up, a ureteric stricture was observed in one (0.80) patient.

Conclusions: The present study suggests that the use of TFL in fragmentation mode, with a power setting of 6 watts and not exceeding 10 watts, is effective. TFL is a safe and effective treatment modality for ureteral laser lithotripsy.

## Introduction

Ureteroscopy is commonly performed to treat ureteral stones. It has a distinct advantage over shockwave lithotripsy in achieving higher stone-free rates in a single procedure and is therefore recommended by the European Urology Association guidelines [1]. Stone lithotripsy can be performed during ureteroscopy by using pneumatic or laser technology. A significant disadvantage of pneumatic lithotripsy is stone retropulsion into the kidneys, which often necessitates an ancillary procedure [2].

Holmium lasers have gained widespread acceptance for stone lithotripsy in both the kidney and ureter and are currently considered the gold standard [3]. Its main advantages include effective stone disintegration, reduced retropulsion, and compatibility with flexible ureteroscopes [4]. However, holmium laser systems have certain limitations, including suboptimal laser beam focusing, low frequency, high cost, and relatively bulky equipment [5].

Since their introduction in 2018, thulium fiber lasers (TFLs) have gained increasing popularity for use in stone lithotripsy. In vitro studies comparing holmium laser and TFL have demonstrated a distinct advantage of TFL, including improved dusting and fragmentation properties, up to fourfold higher ablation rates, and significantly reduced stone retropulsion [6,7]. It has also been observed that the learning curve with TFL is shorter than that with holmium laser, especially among junior urologists [8]. Additionally, the risk of laser fiber fracture is lower with TFLs than with holmium:YAG (Ho:YAG) lasers [9].

A major concern with the use of holmium and TFL lasers during intracorporeal lithotripsy is heat generation, which may cause thermal injury to surrounding tissues. This concern is more pronounced in the ureter than in the kidney because of its smaller working space, limited irrigation, and reduced visibility. This can increase the incidence of ureteral stricture. Therefore, the present study aimed to evaluate the safety and efficacy of TFL in the ureter during the treatment of ureteric stones using semi-rigid ureteroscopy.

## Materials and methods

This retrospective study was conducted in the Department of Urology of our hospital between January 2023 and July 2024. Institutional Ethics Committee approval was obtained from the hospital ethics committee (approval number: ECRHS/SUH/03/2022) before the commencement of the study.

We evaluated 124 consecutive patients who underwent semi-rigid ureteroscopy for ureteric stones at all locations using TFL lithotripsy. Patients with renal or ureteric stones, pyonephrosis, pregnancy, or ureteric strictures were excluded from this study. Preoperative urine culture, sonography, and non-contrast CT data were reviewed. The stone size was noted in the maximum dimension in millimeters, and the stone volume was calculated using CT scan software. The stone location, number, and Hounsfield Unit (HU) values were recorded.

Operative procedure and laser parameters

All procedures were performed under spinal anesthesia by a single surgeon (ADM). After placing the patient in the lithotomy position, a Terumo^TM^ glidewire (Terumo Corporation, Tokyo, Japan) was placed in the kidney under fluoroscopic guidance. Semi-rigid ureteroscopy was performed using a 6.5/7.5 F ureteroscope (Karl Storz, Tuttlingen, Germany). Stone lithotripsy was performed using a TFL (Urolase SP, IPG, Photonics, Moscow, Russia). A 400-µm laser fiber was used in all the procedures.

Laser lithotripsy was performed using either fragmentation or dusting modes. In fragmentation mode, the laser setting included an energy of 1 J with frequencies of 6, 8, or 10 Hz. In the dusting mode, the laser settings were 0.1 J of energy and a frequency of 60 Hz, with a total power of 6 W, and the fragmentation mode using high energy and low frequency was preferred over the dusting mode. In both modes, the total laser power did not exceed 10 W. Operative time was calculated from the start of cystoscopy until the insertion of the double-J (DJ) stent. Laser activation time and total laser energy delivered were recorded for all patients.

Intra- and postoperative findings

Intraoperative visibility was graded by the operating surgeon using a Likert scale and categorized into three grades: grade 0, good vision; grade 1, average vision; and grade 2, poor vision, requiring temporary cessation of lithotripsy until vision improved. Intraoperative bleeding and incidence of stone retropulsion were documented. At the end of the procedure, stone clearance was confirmed under fluoroscopic guidance and by endoscopic inspection of the entire ureter. A DJ stent was placed in all the patients.

Follow-up ultrasonography and plain radiography of the kidneys, ureters, and bladder (KUB) were performed at 15 days to assess stone-free status. The DJ stent was removed 15 days after confirmation of stone clearance. All patients were followed up for three months using repeat ultrasonography and radiography.

Intraoperative complications were classified according to the modified Satava classification of ureteroscopic classification. Postoperative complications, including fever, bleeding, and ureteral stricture, were recorded and graded according to the Clavien-Dindo classification system.

The stone ablation efficacy was calculated as the total energy used (J) divided by the stone volume (mm^3^). The stone ablation speed was calculated as the stone volume (mm^3^) divided by the laser time (s).

Statistical analysis

Statistical analyses were performed using IBM SPSS Statistics for Windows, Version 20 (Released 2011; IBM Corp., Armonk, New York, United States). Continuous variables are expressed as mean and standard deviation (SD), and categorical variables are summarized as frequencies and percentages. No inferential statistical tests were performed because the analysis was limited to descriptive statistics and frequency distribution.

## Results

The ages of the patients ranged from 7 to 72 years. The mean stone size and stone volume were 10.2 mms and 237.74 mm^3^, respectively, with a mean HU value of 962.22. Most stones were located in the lower ureter (62, 49%), followed by the upper ureter (32, 25%), midureter (18, 14.28%), and multiple ureteral locations (14, 11%) (Table 1).

**Table 1:** Patient demographics and stone characteristics Data presented as mean (SD), unless otherwise specified.

Parameters	No. of patients (N=124)
Age (years)	40.27 (13.99)
Gender, n (%)	
Male	98 (79.0)
Female	26 (21.0)
Serum creatinine (mg/dL)	1.35 (1.15)
Urine culture (%)	
Sterile	93 (75)
Escherichia coli	24 (19.35)
Klebsiella	03 (02.41)
Candida	02 (01.61)
Pseudomonas aeruginosa	02 (01.61)
Size of the stone (mm)	10.2 (3.72)
Volume of the stone (mm^3^)	237.74 (344.19)
Hounsfield Unit (HU) of the stone	962.22 (401.33)
Location of stone, n (%)	
Upper	32 (25.39)
Mid	18 (14.28)
Lower	62 (49.20)
Multiple locations	14 (11.11)

The mean operative time was 66 minutes. Intraoperative bleeding was observed in only two (1.61%) patients. According to the Likert scale, intraoperative visibility was graded as good in 94 (75.8%), average in 25 (20.1%), and poor in five (4%) patients. Stone retropulsion occurred in four (3.22%) patients (4/124) (Table 2). Retropulsion was observed in two of the four patients who used TFL in the dusting mode at 60 Hz. In contrast, retropulsion occurred in only two patients when TFL was used in the fragmentation mode.

**Table 2:** Intraoperative parameters Data presented as n (%), unless otherwise specified.

Parameters	No. of patients (N=124)
Surgery time (min), mean (SD)	66.92 (14.88)
Intraoperative bleeding, n (%)	
Yes	2 (01.61)
No	122 (98.38)
Intraoperative vision, n (%)	
Good	94 (75.80)
Average	25 (20.16)
Poor	5 (04.03)
Stone retropulsion, n (%)	
Yes	4 (03.22)
No	120 (96.77)

The TFL was used predominantly in fragmentation mode (120 patients) compared to dusting mode (four patients). In the fragmentation mode, the most commonly used laser setting was 1 J at 6 Hz (total power of 6 W) in 89 patients, followed by 1 J × 8 Hz in six patients and 1 J × 10 Hz in 25 patients. The dusting mode was used in only four patients, with laser settings of 0.1 J X 60 Hz and a maximum total power of 6 W. The mean laser activation time was 11.83 minutes, and the mean total laser energy delivered was 1421 mJ. Stone ablation efficacy was 9.32 J/mm^3^, and stone ablation speed was 0.23 mm^3^ (Tables 3, 4).

**Table 3:** Distribution of laser parameters (N=124) Data presented as n (%).

Energy setting	Frequency (Hz)	n (%)	Total power (Watts)
1J (N=120)	6	89 (71.77)	89 (71.77)
	8	6 (4.84)	6 (4.84)
	10	25 (20.16)	25 (20.16)
0.1 J (N=4)	60	4 (3.23)	4 (3.23)

**Table 4:** Laser performance parameters Data presented as mean (SD), unless otherwise specified.

Laser parameters	Mean (SD)	Range
Laser time (min)	11.83 (6.94)	2.40-39.00
Laser energy (J)	1421.53 (1730.46)	190.00-7840.00
Stone ablation efficacy (J/cubic mm)	9.32 (9.07)	0.379-75.40
Stone ablation speed (cubic mm/seconds)	0.23 (0.19)	0.03-6.90

Intraoperative ureteroscopy-related complications were classified using a modified Satava classification system. Grade I complications (events without consequences) occurred in two (1.61%) patients, Grade IIa complications (requiring intraoperative endoscopic intervention) in four (3.22%) patients, and Grade IIb complications (requiring endoscopic re-treatment) in one (0.80%) patient. Grade III complications were not observed.

Postoperatively, hematuria was observed in 10 patients (8%) and was self-limiting, requiring no intervention. Fever occurred in six patients (4%) and urosepsis in three patients (2.4%). All infectious complications were managed conservatively with appropriate antibiotics, based on urine culture sensitivity. None of the patients required postoperative admission to the intensive care unit (Table 5).

**Table 5:** Postoperative parameters Data presented as n (%).

Complications	No. of patients (N=124)
Postoperative hematuria	10 (08.06)
Fever	06 (04.83)
Urosepsis	03 (2.41)
Ureteric structure	01 (0.80)
Residual stone	
<4 mm	02 (1.61)
>4 mm	02 (1.61)
Modified Satava classification	
Grade I	02 (1.61)
Grade IIa	04 (3.22)
Grade IIb	01 (0.80)
Grade III	0
Clavien-Dindo classification	
I	12 (9.67)
II	03 (2.41)
III	1 (1.61)
IV	-
No	107 (86.29)
Stone free	122 (98.39)

According to the Clavien-Dindo classification, postoperative complications were Grade I in 12 patients (9.67%), Grade II in three patients (2.4%), and Grade III in one patient (1.6%). On long-term follow-up, ureteric stricture was observed in only one patient (0.8%). The stone-free status was assessed using ultrasonography and radiography. Four patients had residual stones; of these, two had residual fragments <4 mm that passed spontaneously, whereas two had residual stones >4 mm that were removed during DJ stent removal. The overall stone-free rate was 98% (Table 5).

## Discussion

This study evaluated 124 patients who underwent semi-rigid ureteroscopy with TFL for stone lithotripsy. TFL lithotripsy results in improved intraoperative visualization, low stone retropulsion rates, favorable laser ablation efficacy and speed, and minimal complications. The Ho:YAG laser is widely used and considered the gold standard for stone lithotripsy because it effectively fragments all stone types [4]. However, Ho:YAG lasers also have several limitations. Endoscopic visibility may be compromised due to the “snowstorm” effect during lithotripsy; smaller fibers may not efficiently transmit laser energy, resulting in prolonged operative time, and stone retropulsion is commonly observed during clinical use [10]. Additional limitations include the need for a cooling mechanism, which increases the machine size and cost, possible mirror misalignment due to external impact leading to inefficient laser delivery, requirement for larger fibers, and relatively poor dusting efficiency [11]. In contrast, TFL offers distinct advantages, including superior dusting and fragmentation properties, up to four-fold higher stone ablation rates, and significantly reduced retropulsion compared to Ho:YAG lasers [7].

In the ureter, where the operative field is limited, maintaining adequate intraoperative visualization during laser lithotripsy is crucial for preventing ureteral mucosal injury and subsequent complications. Increasing irrigation to improve vision may increase the risk of infectious complications [12]. Gupta et al. compared intraoperative vision during Ho:YAG and TFL lithotripsy and reported excellent endoscopic visibility in 75% of TFL cases compared to only 25% with Ho:YAG [5]. Similarly, the present study observed good visibility in approximately 75% of the patients undergoing TFL lithotripsy for ureteric stones. This improved visibility is likely attributable to the use of smaller fibers and the reduced snowstorm effect associated with the TFL.

Stone retropulsion is a critical factor during ureteric stone lithotripsy as it may necessitate an ancillary procedure. Enikeev et al. demonstrated a strong correlation between laser visibility and pulse energy, and an increased incidence of retropulsion [13]. Martov et al. reported no retropulsion with TFL compared to a 31% retropulsion rate in the Ho:YAG group [10]. In this study, retropulsion occurred in only four patients (3.2%). Of these, two stones were managed with flexible ureteroscopy in the same session, while the remaining two patients passed small residual fragments. The low retropulsion observed with the TFL may be explained by the formation of smaller vapor bubbles that collapse with less energy at the fiber tip [6,14]. Additionally, the longer pulse duration and lower peak power of TFL reduce the magnitude and initial velocity of retropulsion [15,16].

Enikeev et al. suggested two laser settings for ureteroscopic stone lithotripsy with TFL. First, the laser was used in fragmentation mode at 0.5 J, 30 Hz for a total power of 15 W. This can be reduced to 12 Hz to increase visibility. The second was low energy and high frequency (0.15 J x 100 Hz) in dusting mode [13]. Several other in vitro studies have used TFL in the high-frequency mode [6,17]. Based on our clinical experience, we recommend using TFL in the fragmentation mode with a laser setting of 1 J × 6 HZ (6 W), which is sufficient in most ureteric stone cases. For larger or more complex stones, the frequency can be increased to 10 Hz to reduce laser exposure time. We do not recommend exceeding the total power of 10 W in the ureter. High-frequency modes are avoided in the ureter because maintaining stable contact of the fiber with the stone is difficult, thereby increasing the risk of ureteral mucosal injury.

Several studies have demonstrated improved stone-free rates with TFL compared with Ho:YAG laser [10,17]. In a randomized controlled trial, Ulvik et al. reported stone-free rates of 86% for renal stones and 100% for ureteral stones by using TFL [18]. Mahajan et al. compared pneumatic lithotripsy and TFL for ureteric stones and observed stone-free rates of 94% with TFL and 92% with pneumatic lithotripsy at seven days [19]. In our study, the immediate stone-free rate was 98%, which was comparable to the rates reported in the literature.

Thermal injury during an intracorporeal lithotripsy remains a concern. Molina et al. studied the temperature profiles of high-power Ho:YAG and TFL in ex vivo porcine kidneys and found comparable intraluminal temperature increases in the dusting mode, with higher temperatures observed during fragmentation mode with TFL; however, thermal injury thresholds were not reached with either laser [20]. Belle et al. reported higher heat generation with TFL in most settings, although at 20 W, temperatures were comparable between TFL and Ho:YAG lasers [21]. As the temperature rise is directly proportional to the laser power, many authors recommend limiting the laser power to 20-30 W in the kidney and 15-20 W in the ureter to avoid thermal cellular damage [22,23]. In the current study, the total power was restricted to ≤10 W, with most patients treated at 6 W. Adequate irrigation, which is critical for minimizing thermal injury, was maintained throughout the procedure [24,25]. Sierra et al. demonstrated that with continuous irrigation at a water pressure of 40 cm, intraluminal temperatures remained within safe limits for both TFL (36.8°C) and Ho:YAG (34.6°C) lasers [8].

Laser-induced thermal injury can lead to ureteric strictures. Ahmad Para et al. reported a higher stricture rate with TFL, which was attributed to the increased tissue absorption and higher temperatures [26]. Given its narrow ureteral lumen and limited irrigation, meticulous laser use is essential. Several studies have shown improved irrigation and visibility with TFL [5,13]. Enikeev et al. reported a reduced risk of ureteral injury when adequate irrigation was maintained, and the laser-tissue distance exceeded 2 mm [13]. In the present study, good intraoperative visibility likely contributed to the low stricture rate; only one patient developed a distal ureteric stricture, which was successfully managed with DJ stenting.

The main limitations of this study are its retrospective design and lack of a comparative arm. Temperature monitoring was not performed to evaluate its effect on the ureteric mucosa. Stone-free rates and postoperative complications were evaluated using ultrasonography and X-ray KUB, with CT scans reserved for symptomatic patients or for those with worsening hydronephrosis.

## Conclusions

The findings of this study demonstrate that TFL use in low-power settings of 1 J x 6 Hz (power = 6 W) in the ureter, not exceeding 10 W, is safe and effective for laser lithotripsy during semi-rigid ureteroscopy in the management of ureteric stones.
